# Annual Accumulation of CymMV May Lead to Loss in Production of Asymptomatic Vanilla Propagated by Cuttings

**DOI:** 10.3390/plants13111505

**Published:** 2024-05-30

**Authors:** Fenglin Gu, Fei Xu, Guiping Wu, Hongying Zhu, Changmian Ji, Yu Wang, Qingyun Zhao, Zhiyuan Zhang

**Affiliations:** 1 Sanya Research Institute of Chinese Academy of Tropical Agricultural Sciences, Sanya 572025, China; xiaogu4117@catas.cn (F.G.); jichangmian@itbb.org.cn (C.J.); wangyu@itbb.org.cn (Y.W.); 2 Spice and Beverage Research Institute, Chinese Academy of Tropical Agricultural Sciences, Wanning 571533, China; xufei_0302054@163.com (F.X.); guiping81@163.com (G.W.); zhhy-2004@163.com (H.Z.); 3 Hainan Institute, Zhejiang University, Sanya 572025, China

**Keywords:** *Vanilla planifolia* Andrews, glucovanillin, transcriptome, *Cymbidium mosaic virus*

## Abstract

Vanilla (*Vanilla planifolia* Andrews) is a valuable orchid spice cultivated for its highly priced beans. Vanilla has been planted in Hainan province of China via cutting propagation for about 40 years. The yield has been decreasing annually for the past ten years due to pod numbers declining significantly even though it seems to grow normally without disease symptoms, while the reason is still unknown. In this study, we found that *Cymbidium mosaic virus* (CymMV), one of the most devastating viruses causing losses in the vanilla industry, massively presented within the pods and leaves of vanilla plants, so the virus infecting the vanilla seems to be a highly probable hypothesis of the main contributions to low yield via decreasing the number of pods. This represents the first speculation of CymMV possibly affecting the yield of vanilla in China, indicating the important role of virus elimination in restoring high yield in vanilla. This research can also serve as a warning to important economic crops that rely on cuttings for propagation, demonstrating that regular virus elimination is very important for these economically propagated crops through cuttings.

## 1. Introduction

Vanilla is a tropical orchid, known as the “king of food and spices” [1], which is the second most expensive spice in the world and is one of the most important and popular aromatic compounds used in food, beverages, and cosmetics [2]. In Vanilla, Vanillin (3-methoxy-4-hydroxybenzaldehyde) and its derivatives are the most important and economically valuable compounds. Although vanillin originates from the pods of Vanilla, its production process is quite complex, because a high concentration of vanillin is toxic to living cells. In the fresh pod of vanilla, vanillin is produced and stored as non-toxic glycovanillin, and at this stage, the beans exhibit no trace of vanilla flavor. During the curing process of vanilla beans, glycovanillin undergoes transformation into vanillin when it encounters the enzyme of β-glucosidase. This enzyme catalyzes the hydrolysis of glycovanillin, effectively cleaving the glucose molecule attached to the vanillin structure. As a result, free vanillin is released, contributing to the characteristic aroma and flavor of cured vanilla [3]. The flower of vanilla is yellow, bisexual and develop towards the top of the plant when the vine is approximately 4–5 m long. When successful pollination occurs, each flower yields a single pod [4], and 8–9 months after pollination (DAP), the vanilla pods are usually harvested and processed by curing to generate the vanillin [5]. Due to the long production cycle and complex processing, natural vanillin is limited in quantity and expensive. Vanilla have successfully adapted to new geographies that are now recognized as today’s major vanilla-growing regions, including Madagascar, Indonesia, Uganda, India, and other areas [6]. Today, most vanilla-growing regions rely on vegetative propagation of vanilla to increase planting material. Consequently, plants used in commercial production are largely genetically identical to their original wild clone. This propagation method puts vanilla plants at high risk for viral infection, as viruses can easily be transmitted through this process.

Viral diseases have been reported in most vanilla-producing countries around the world. Now, at least ten viruses from various genera and families have been identified as infecting vanilla globally [7,8,9]. Among them, *Cymbidium mosaic virus* (CymMV) belonging to the family *Alfaflexiviridae* is one of the most widespread virus and is known to infect *Vanilla planifolia* and *Vanilla tahitensis* in French Polynesia and Reunion Island [8]. CymMV infection in orchids can be detected from the symptoms such as mosaic patterns, flowers with deformities and colour breaking, sunken chlorotic or necrotic patches on leaves and stems [10,11,12]. 

CymMV has flexuous filamentous particle morphology with a single species of linear positive sense single stranded RNA. The first complete sequence of CymMV genome came from Singapore [10,13]. The nucleic acid length of genome is about 6 kb coding for five proteins, including the RNA-dependant RNA polymerase (*RdRp*), triple gene block 1 (*TGB1*), triple gene block 2 (*TGB2*), triple gene block 3 (*TGB3*) and Coat protein (*CP*) [14]. The infection of vanilla by CymMV often accompanied by chlorotic flecks on leaves and reduces plant vigor and lower flower quality [15]. It sometimes caused no symptom on vanilla according to previous reports [7,16]. Although viruses can infect plants with no apparent symptoms, they can still result in plant yield loss [17]. However, the impact of this phenomenon on vanilla has not yet been evaluated.

The yield of vanilla has been observed to correlate with the agricultural practices employed in field planting as well as the specific years of cultivation. Our observations indicate that the production of vanilla pods surpassed the 500 kg per hectare prior to the year 2013. However, in this study, we found that even with the same planting management techniques and planting years of vanilla, the production of vanilla has been declining for the past ten years. In the present work we describe the massive presence of CymMV existing in plants of vanilla, which might provide a speculative explanation of the decline in vanilla yield, potentially due to reduced flowering.

## 2. Results and Discussion

Vanilla orchids are perennial plants that are propagated through cuttings. They typically begin to flower and bear fruit after two years of cultivation, and reach their peak production period after 5–6 years. Thereafter, as the cultivation period increases, the yield gradually declines. Therefore, it is necessary to observe the growth and monitor the yield every year. When the yield is very low, carrying out replanting is needed.

### 2.1. Significant Decline in Vanilla Pod Yield over the Past 10 Years

**Figure 1:**
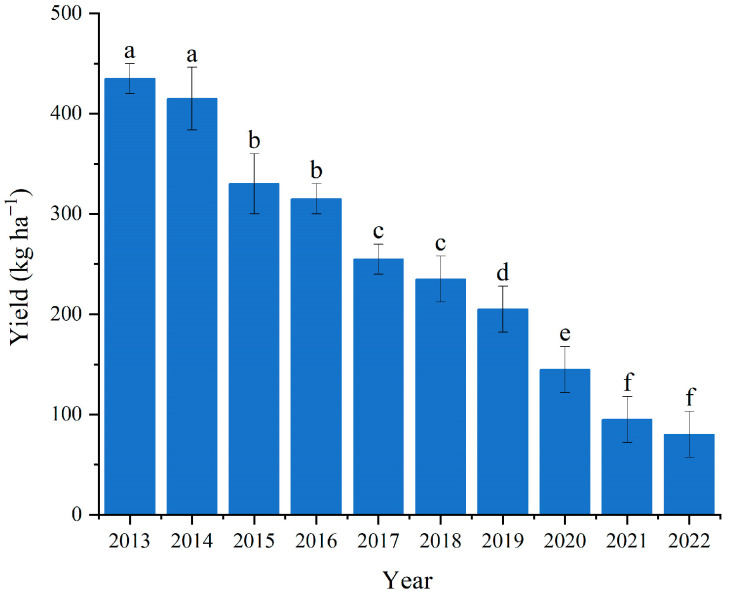
Change in vanilla pods yield harvested from the field in different planting years. Vertical bars are the mean ± standard deviation of three replicates. Bars with different letters indicate significant differences (*p* ≤ 0.05) by Duncan’s test.

During the past ten years, the yield of vanilla pods has significantly decreased from 435 kg per hectare to 80 kg per hectare (Figure 1). For instance, yields of vanilla pods harvested in 2016, 2019, and 2022 decreased by 27.6%, 52.9%, and 81.6% compared to 2013, respectively. Observations of vanilla plantation over the previous decade indicate minimal changes in the growth of the plants, with water, fertilizer, and climate having little impact, yet there has been a substantial decrease in pod yield. The yield in 2022 was approximately 80% lower than that in 2013 and 2014. 

### 2.2. The Process of Planting, Growing, and Harvesting Vanilla in the Fields

**Figure 2:**
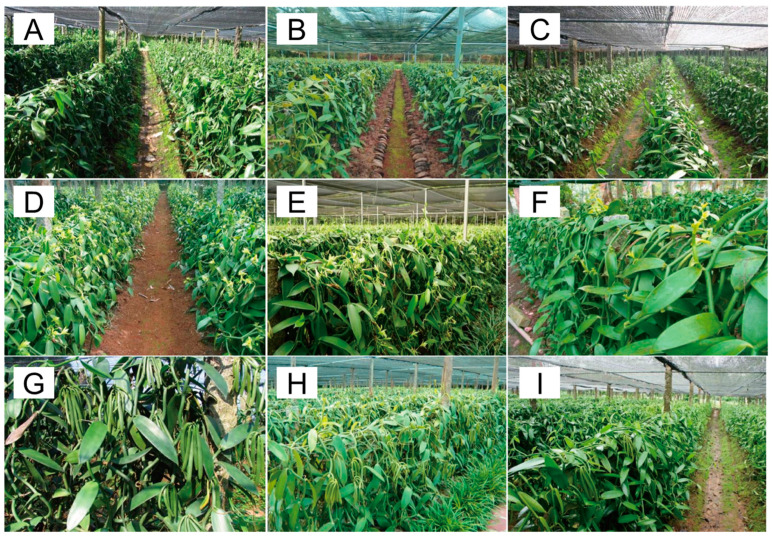
Growth conditions (Vegetative period, flowering period, and fruiting period) of vanilla plantation with six years planting history in 2013, 2018 and 2022, respectively. (**A**–**C**) represent the vegetative period of vanilla plantation in 2013, 2018, and 2022, respectively; (**D**–**F**) represent the flowering period of vanilla plantation in 2013, 2018, and 2022, respectively; (**G**–**I**) represent the fruiting period of vanilla plantation in 2013, 2018, and 2022.

**Figure 3:**
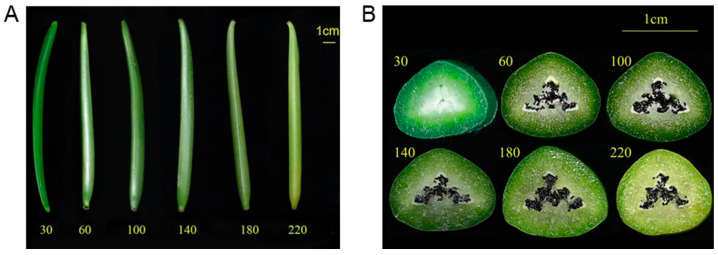
Vanilla fruits at different stages of development and maturation. Representative photographs (**A**) and transverse section (**B**) of vanilla pods at 30, 60, 100, 140, 180 and 220 days after pollination (DAP).

Three vanilla plantations with six-year planting history were selected to assess the growth conditions in 2013, 2018, and 2022, respectively. All these plantations use identical planting management practices. As shown in Figure 2, while the overall growth of the vanilla plantation remained consistent, significant differences were observed in the number of flowers and pods. The plantation had the most flowers and fruits in 2013, while less in 2018, and the lowest flowering and fruit occurs in 2022. To characterize the variability in color, shape, and biochemical components of pods, we sampled pods at six post-anthesis stages. Vanilla pods without mechanical damage and free of visible defects or decay were collected at 30, 60, 100, 140, 180, and 220 days after pollination (DAP) in the year of 2022. For each time-series sample, six pods were randomly collected and immediately transported to the laboratory on ice. There were clear differences in shape, size, and color during pods development (Figure 3A). The pods initially appear dark green at 30 days after pollination (DAP), progressively lighten in color, and turn a light yellowish-green upon reaching maturity. Although the length of pods remains relatively stable from 30 DAP to maturity, they attain full size approximately 45 days after pollination in Hainan province, China.

The transverse section of the pods in vanilla could divide into three visually distinct areas: the outer part (greener area), inner part (white/yellowish green area) and the seeds. The outer part consists of the epicarp and outer mesocarp, while the inner part includes the inner mesocarp, placental laminae, endocarp, and seeds [4]. The mesocarp comprises 15–20 layers of large cells. The seeds are positioned towards the cavity of the pod (Figure 3B) [4,17]. At 30 DAP, the seeds have not yet formed, and the central region of the pod prominently features white placentae. At 60 DAP, the seeds are clearly black, and as time progresses, the placentae decrease significantly while the seeds become more clustered (Figure 3B).

Vanilla pods are typically ready for harvest in six to nine months after pollination. According to our experience, the vanilla pods can be harvested approximately seven months post-pollination in China. The pods are picked individually once fully grown and beginning to ripen, changing color from dark green to light green with yellow tinge. Harvesting immature pods results in an inferior product, while overly ripe pods may start to split. Therefore, it is crucial to monitor the plantation frequently, ideally two to three times a week, to ensure pods are picked at the optimal stage. Green pods naturally lack aroma. Processing and curing should begin within a week of harvesting. The primary cue for harvest readiness is the yellowing of the pod’s apex, signaling full maturity. Initially odorless, the pods require further processing or curing to develop their characteristic aroma, primarily through the activation of precursors-glycovanillin, which is a key component in the aroma profile of processed vanilla pods.

### 2.3. Glucovanillin Accumulates Normally in Vanilla Pods

**Figure 4:**
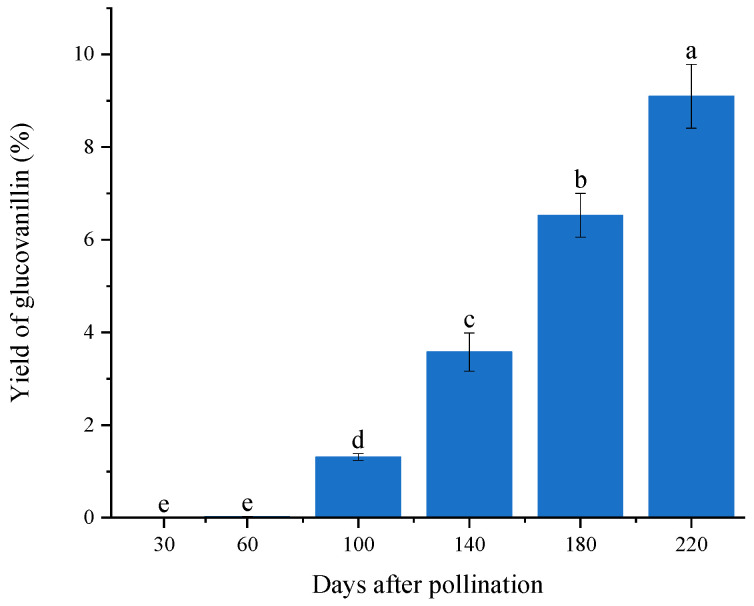
Glucovanillin contents dynamics in vanilla pods after pollination. Vertical bars are the mean ± standard deviation of three replicates. Bars with different letters indicate significant differences (*p* ≤ 0.05) by Duncan’s test.

To monitor whether glucovanillin accumulates normally and analyze its accumulation pattern, we conducted glucovanillin measurements on pods at different stages. As shown in Figure 4, glucovanillin is not detectable in vanilla pods sampled at 30 DAP, but it is present in pods at 60 DAP, with its content increasing significantly thereafter. The glucovanillin contents in vanilla pods are 1.31%, 3.58%, 6.53%, and 9.10% at 60, 100, 140, 180, and 220 DAP, respectively. The highest glucovanillin content is found in mature green pods, while the lowest is in tender young pod, even those of the same length where the placentae and seeds have already formed. The accumulation of glucovanillin progresses gradually as the pods mature [18].

### 2.4. CymMV Massively Accumulates in Pods and Leaves of Vanilla

**Figure 5:**
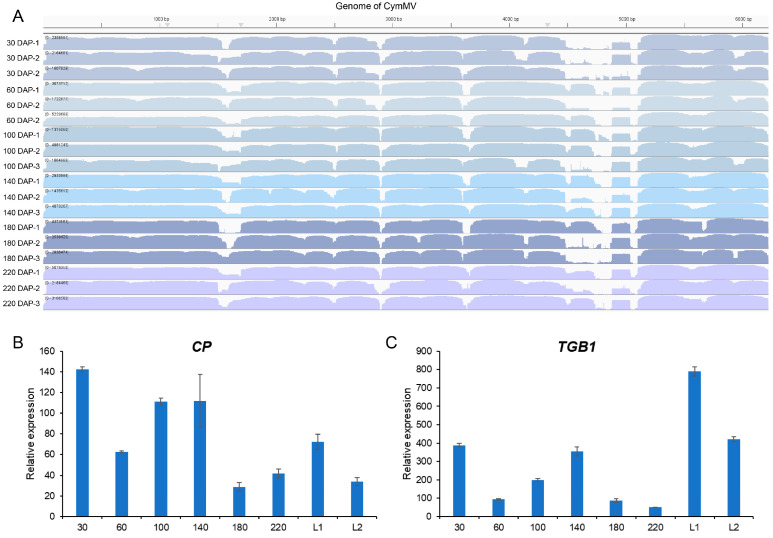
Detection of CymMV in the pods and leaves of vanilla plants. (**A**) This description outlines a read coverage map of CymMV as detected by RNA-seq, analyzed using CLC Genomics Workbench v23.0.2. The heatmap, color-coded to depict varying coverage levels, displays the number of reads. The y-axis quantifies the reads that correspond to reference genomes (GenBank Accession number U62963). (**B**,**C**) qRT-PCR analysis of CymMV in pods and leaves of Vanilla. CP and TGB1 is the coat protein and triple gene block 1 of CymMV. 30, 60, 100, 140, 180, and 220 represent the pods of 30 DAP, 60 DAP, 100 DAP, 140 DAP, 180 DAP, and 220 DAP. L1 and L2 represent the young and mature leaves of Vanilla. Three biological replicates were performed per reaction, each with two technical replicates (using the same sample).

Vanilla pods were collected for transcriptome sequencing at 30, 60, 100, 140, 180, and 220 DAP to detect the presence of viruses in the vanilla pods. Surprisingly, our transcriptome data showed that a virus, named CymMV, accounted for 53–88% of the transcriptome reads (Figure 5A; Table S1), suggesting a substantial viral presence in the pods. Subsequently, we confirmed the RNA-seq results using quantitative Reverse Transcription Polymerase Chain Reaction (qRT-PCR). The CymMV virus contains five genes, and we used the coat protein (*CP*) and triple gene block 1 (*TGB1*) of CymMV as markers to detect the virus in the pods and leaves of vanilla. The results indicated the presence of CymMV in both the leaves and pods, with its transcripts being several hundred times that of the internal control gene of the vanilla, proving a high viral load (Figure 5B,C). Based on the discovery of the massive virus in plants of vanilla, we strongly suspect that it may be impacting pod yield. Vanilla is propagated by cuttings, and the virus can be transmitted from mother plant to its clones. Over time, this may lead to a gradual accumulation of the virus, significantly reducing both flowering and fruit production. In response, we will implement virus elimination treatments and conduct subsequent experiments to validate our hypothesis. 

Interestingly, similar to our findings, it has been observed in strawberries that after infection by the strawberry virus 1, the fruit size remains unchanged and the plants do not display any symptoms. However, there is a significant reduction in the number of flower clusters, leading to a decrease in strawberry yield [19]. This aligns with our report that after significant accumulation of the virus in vanilla, despite showing no symptoms, the reduction in flower number leads to decreased yield. Therefore, for crops propagated by cuttings, a reduction in yield may serve as an indicator of viral accumulation within the plant, and virus elimination treatments can be carried out promptly to rule out the possibility of viral accumulation, regardless of symptom presentation.

## 3. Materials and Methods

### 3.1. Materials

Vanilla pods without mechanical damage and free of visible defects or decay were collected at 30, 60, 100, 140, 180, and 220 DAP from Spice and Beverage Research Institute of Chinese Academy of Tropical Agricultural Sciences, Xinglong, Hainan Province, China (110°20′ E, 18°73′ N). The region is characterized by a mean annual temperature of 24.5 °C and an average annual precipitation of 2201 mm. 

For each time-series sample, six pods were randomly collected and immediately transported to the laboratory on ice. After being cut with a sterilized knife, a portion of about 1 cm in length from the middle of the pods was selected for photographing the cross section. The other portions were immediately frozen in liquid nitrogen and stored at −80 °C for subsequent DNA and RNA extraction. The last portions of the six pods were freeze-dried, mixed, and smashed to determine the content of glucovanillin triplicately. Methanol (MeOH), acetonitrile (ACN) and ethanol for LC-MS analysis (chromatographic purity grade) were purchased from Merck. Other chemicals for extraction analysis (analytical reagent grade) were purchased from Shanghai Chemical Reagent Co., Ltd. (Shanghai, China).

### 3.2. Growth Condition and Anatomical Observations

Photographs documenting the plant growth conditions in the plantation were taken using a Nikon Z7 digital camera (Tokyo, Japan). Fresh vanilla pods were immediately rinsed with water and surface moisture was dried. They were then longitudinally sliced open at the center using a scalpel. 

### 3.3. Vanilla Yield in Field Condition

Field experiments were conducted on a 10 m × 30 m area, which was evenly divided to three plots, each measuring 10 m × 10 m and planted with 100 plants, and the three plots served as the replicates. Various vanilla plantation with 6 years of planting history were selected to investigate the yields from 2013 to 2022. Vanilla pods were harvested annually in November to obtain fresh weight.

### 3.4. Determination of Glucovanillin

Glucovanillin was extracted by 2 mL of a 50:50 methanol:water solution (*v*/*v*) for 2 h at ambient temperature, with intermittent sonication. The mixture was then centrifuged at 14,000× *g* for 5 min, and the supernatant underwent quantitative analysis. Glucovanillin concentrations were determined in triplicate using high-pressure liquid chromatography (HPLC;1260 series HPLC; Agilent, Santa Clara, CA, USA), following the slightly modified method of Dong et al. [20]. The standard of glucovanillin was purchased from Sigma Chemical Company (St. Louis, MO, USA). The HPLC was equipped with a Zorbax Eclipse Plus C18 column (4.6 mm by 100 mm; particle size, 3.5 m; Agilent). Isocratic elution was carried out at a flow rate of 1.0 mL/min using a mixture of 20% methanol and 80% acidified water. Detection was conducted with a UV detector (HPLC;1260 series HPLC; Agilent, Santa Clara, CA, USA) set at 280 nm, while the column temperature was maintained at 26 °C.

### 3.5. Transcript Analysis

30, 60, 100, 140, 180, and 220 DAP of pods with three replications were collected and subjected to RNA isolation and purification using the RNAprep Pure Plant Plus kit (TIANGEN BIOTECH (BEIJING) Co., Ltd., Beijing, China). Total RNA quality was assessed using the Fragment Analyzer 5400 (Agilent Technologies, CA, USA). cDNA libraries were constructed using NEBNext^®^ UltraTM RNA Library Prep Kit for Illumina^®^ (New England Biolabs, Massachusetts, USA) and subjected to sequencing. The library formulation was sequenced using the Illumina Novaseq 6000 platform to generate paired-end reads of 150 bp. Raw data converted from the Illumina platform was subjected to quality control before subsequent analysis. Randomly extract 10,000 reads from the filtered high-quality data and align them to the National Center for Biotechnology Information Taxonomy Database using the Blast software.

### 3.6. qRT-PCR Analysis

Total RNA was extracted from 30, 60, 100, 140, 180, and 220 DAP of pods and leaves. HiScript IIQRT SuperMix from Vazyme was used to synthesize first-strand cDNA. qRT-PCR was performed with an ABI 7500 real-time PCR system using ChamQ Universal SYBR qPCR Master Mix from Vazyme. The code sequence of coat protein (*CP*) and triple gene block 1 (*TGB1*) of CymMV (Genebank: U62963) was used to design primers for the qRT-PCR experiment using the online website Primer-Blast (https://www.ncbi.nlm.nih.gov/tools/primer-blast/; accessed on 12 July 2023). Vanilla *actin 1* gene was used as an internal control for normalization between samples [21]. Relative transcript levels were calculated using the 2^−ΔΔCT^ method. Primers for qRT-PCR were listed in Table S2.

### 3.7. Statistical Analysis

Duncan test was used to determine statistical significance of all the data except for the correlation analysis. A value of *p* < 0.05 was considered to indicate a significant difference between the groups. In addition, one-way analysis of variance (ANOVA) was utilized for multiple group comparison by using GraphPad Prism Version 6.00. Experimental data are given as mean ± standard deviation. 

## 4. Conclusions

This study presents a detailed analysis of the annual yield of the six-year-old newly transplanted vanilla garden, along with the anatomical morphology and transcriptomic profile of the pods post-flowering. Although the growth of vanilla plants remains basically stable, there is a consistent year-over-year decline in pod yield, even among plantations of the same planting age. The anatomical morphology of the pods indicates that 30 DAP the pod length basically reaches the same length as mature pods, and the internal structure of the pods also basically reaches the same state as mature pods around 30 DAP. However, the key component glucovanillin in the pods is not produced within 60 DAP, but gradually produced after 60 DAP, reaching the highest level when the pods mature. Transcriptomic analysis of the pods at different times after flowering shows a high presence of CymMV, suggesting this could be a highly probable factor in the yearly decline in pod yield. Further experiments will aim to confirm this hypothesis. 

## Data Availability

The original contributions presented in the study are included in the article/Supplementary Material, further inquiries can be directed to the corresponding author.

## Supplementary Materials

The following supporting information can be downloaded at: https://www.mdpi.com/article/10.3390/plants13111505/s1, Table S1: The statistical analysis of the proportion of viral reads to total transcriptome reads. Table S2: Primers for qRT-PCR.
